# Isolation and Characterization of a Novel Endoglucanase from a *Bursaphelenchus xylophilus* Metagenomic Library

**DOI:** 10.1371/journal.pone.0082437

**Published:** 2013-12-27

**Authors:** Lin Zhang, Yongxin Fan, Haoying Zheng, Fengguang Du, Ke-qin Zhang, Xiaowei Huang, Linfeng Wang, Man Zhang, Qiuhong Niu

**Affiliations:** 1 Department of Life Science and Biotechnology, Nanyang Normal University, Nanyang, China; 2 State Key Laboratory of Motor Bio-fuel technology, Henan Tianguan Group Co. Ltd., Nanyang, China; 3 Laboratory for Conservation and Utilization of Bio-Resources, and Key Laboratory for Microbial Resources of the Ministry of Education, Yunnan University, Kunming, China; Rockefeller University, United States of America

## Abstract

A novel gene (designated as *cen219*) encoding endoglucanase was isolated from a *Bursaphelenchus xylophilus* metagenomic library by functional screening. Sequence analysis revealed that *cen219* encoded a protein of 367 amino acids. SDS-PAGE analysis of purified endoglucanase suggested that Cen219 was a monomeric enzyme with a molecular mass of 40 kDa. The optimum temperature and pH for endoglucanase activity of Cen219 was separately 50°C and 6.0. It was stable from 30 to 50°C, and from pH 4.0 to 7.0. The activity was significantly enhanced by Mn^2+^ and dramatically reduced by detergent SDS and metals Fe^3+^, Cu^2+^ or Hg^2+^. The enzyme hydrolyzed a wide range of β-1, 3-, and β-1, 4-linked polysaccharides, with varying activities. Activities towards microcrystalline cellulose and filter paper were relatively high, while the highest activity was towards oat gum. The *K_m_* and *V_max_* of Cen219 towards CMC was 17.37 mg/ml and 333.33 U/mg, respectively. The findings have an insight into understanding the molecular basis of host–parasite interactions in *B. xylophilus* species. The properties also make Cen219 an interesting enzyme for biotechnological application.

## Introduction

Limited fossil resources, growing economies and an everlasting burden on our environment have caused an increasing interest for alternative resources to produce fuels and chemicals [1]. Lignocellulose, composed mainly of cellulose, hemicellulose, and lignin, is the most abundant renewable carbon source on earth and therefore an attractive resource to use it for the production of different chemical building blocks or biofuels [2]. However, due to the compact microfibrils formation and complex crystalline organization together with the heterogeneous polysaccharide network, lignocellulosic biomass is particularly recalcitrant to deconstruction and poorly susceptible to both chemical and enzymatic hydrolysis. Therefore, urgent demands for the degradation of lignocellulose into its component sugars led to an increased interest in effective cellulase research [3]–[5]. In fact, in recent years many important cellulases have been successfully found from a variety of organisms including archaea, bacteria, fungi, plants, and animals and some of them especially fungal cellulases have been used in commercial for biomass conversion of cellulose or hemicelluloses [6]–[12]. Even though, enzyme production costs and complex pretreating lignocellulosic materials with chemicals and/or heat still constitute limiting factors to wide-scale and rapid biomass conversion [6], [13], [14]. Thus, searching novel cellulases from extreme and special environments becomes an attractive alternative.

Perhaps one of the best characterized examples of an effective complex biomass degrading community is that harbored within the pinewood nematode (PWN), *Bursaphelenchus xylophilus* (*Bx*). *Bx* is the pathogenic agent of pine wilt disease (PWD), which has caused serious damage to pine forests in Japan, Korea, and China [15]. Despite the significance of this disease, the pathogenic mechanism of PWD remains unclear. *Bx* infects the above-ground parts of trees and quickly kills its host. Once *Bx* enters the tree, they feed on plant cells in the tree, leading to disruption of pine tissues and lethal wilt. When feeding on plants, *Bx* uses a needle-like feeding structure, the stylet to pierce the cell wall and ingest nutrients from the cytoplasm [16]. The proteins secreted from the stylet are produced in the esophageal glands (subventral and dorsal glands). Furthermore, many proteins also secreted from the hypodermis or released from natural openings of the nematode [17]. These secretions would contain crucial cell-wall degrading enzymes in the interaction of the nematode with its host plant. As a result, we think *Bx* and its associated microbes maybe an ideal and valuable resource for obtaining effective cellulases.

In fact, several cellulase genes have been isolated and characterized from *Bx* and almost all of them were reported as acquired by horizontal gene transfer from bacteria [18]. However, it is generally accepted that a large proportion of the microorganisms (more than 99%) in many complex natural environments are not cultivable [19]–[21]. This unexplored microbial diversity represents an untapped source of potentially novel and unique enzymatic activities and metabolic pathways that can be applied to industrial biomass conversion [20], [22], [23]. The metagenomic strategy could be applied to screen for biocatalysts with novel characteristics for biotechnological applications without requiring the cultivation of microorganisms [24]. Various industrial biocatalysts such as lipase/esterase, amylase protease and tannase have been isolated from metagenomic libraries [25]–[29].

In this work, a *Bx* and associated microbe metagenomic library was constructed for screening cellulase genes. The gene *cen219* encoding endoglucanase was cloned and sequenced. Subsequently, *cen219* was expressed in *Escherichia coli* BL21, purified and characterized. To our knowledge, this is the first report on cellulase gene isolated from *Bx* and associated microbes.

## Materials and Methods

### Ethic Statement

No specific permission was required for the locations. The field studied is state-owned and belongs to a tourist district open to any visitors. We only took *Bx* samples from the infected pine trees and did not involve any endangered or protected organisms. All the studies were performed according to the law of the People's Republic of China.

### Bacterial Strains and Materials

*Eschericia coli* JM109 served as the cloning host (Novagen, Gibbstown, NJ, USA). *E. coli* BL21 (DE3) (Novagen, Madison, USA) was used for protein expression. The pUC118 (TaKaRa, Dalian, China) and pET-30a (t) (Novagen, Madison, WI, USA) were used to construct metagenomic libraries and express the target protein, respectively. *E. coli* transformants were grown at 37°C in Luria-Bertani (LB) broth with appropriate antibiotics. The custom molecular biology reagents were purchased from TaKaRa (Dalian, China).

### DNA Extraction from *B. xylophilus* Samples

*Bx* samples were isolated from WPD epidemic areas in Baotianman Natural Reserve Area in the central region of China. The libraries would represent both the *Bx* and associated microbial DNA. The samples (∼50,000 worms) were washed three times with sterile water followed by grinded up using liquid nitrogen for 10 min. Then the samples were mixed with 20 ml of DNA extraction buffer (100 mM Tris-HCl [pH 8.0], 100 mM sodium EDTA [pH 8.0], 100 mM sodium phosphate [pH 8.0], 1.5 M NaCl, 1% CTAB), 1 mg/ml proteinase K and 1.5 ml of 20% SDS. The mixture was incubated in a 65°C water bath overnight. The supernatants were collected after centrifugation (9,600×g, 10 min) at 4°C and transferred into 50 ml centrifuge tubes. An equal volume of phenol chloroform was added and gently mixed. The aqueous phase was recovered by centrifugation and precipitated with 0.6 volume of isopropanol at −20°C for 3 h. The pellet of crude nucleic acids was obtained by centrifugation (9,600×g, 20 min) at 4°C, washed twice with cold 75% ethanol, suspended in an appropriate volume of sterile deionized water and stored at −20°C.

### Construction of a Metagenomic Library

The metagenomic library was constructed based on a method described previously with minor modifications [29]. The DNA was purified using β-Agarase and partially digested with *Bam*HI. The digestion product was controlled in 2.5–10 kb size range. After purification, the DNA fragments were ligated into *Bam*HI-digested pUC118, and the ligated products were transformed into *E. coli* JM109. The transformed cells were plated onto LB agar plates containing 100 µg/ml ampicillin, 0.5 mM isopropyl-β-D-thiogalactopyranoside (IPTG) and 40 µg/ml 5-Bromo-4-chloro-3-indolyl β-D-galactopyranoside (X-gal). Then clones with white color were selected for screening those with cellulase activities.

### Screening for clones expressing cellulase activities

To obtain the transformants with cellulase activity, the method described previously was used as the screening way with minor modifications [30]. The cultures selected above were replicated into LB agar with 100 µg/ml ampicillin using replica plating methods. The plates were incubated at 28°C for 4 days. Afterwards, the strains were assayed for their ability to degrade CMC by incubation with 0.1% congo red solution for 30 minutes followed by washing with 5 M NaCl. All the strains with a clear zone around the colonies were chosen as positive. A comparison of the cellulase production was then carried out by agar spot method according to the literature [31]. Firstly, the tested bacterial suspensions were adjusted to the same turbidity by comparison with McFarland Turbidity Standard at the value 0.5 (corresponding to about 1.5×10^8^ CFU ml^−1^), in 25 ml of Ringers solution (Sigma-Aldrich). Then the same amount of cells was spotted on agar medium in triplicate. After incubation at 28°C for 4 days, the spots were stained with 0.1% congo red and the dimensions of the clearing zones were measured from the border of the colony to the outer edge of the zones. Experiments were performed in duplicate.

### Sequencing and analysis of cellulase genes

The clone with the maximum clearing zones was selected for DNA sequencing using a BigDye sequencing kit and ABI 377 DNA sequencer. The deduced amino acid sequence analysis and open reading frame search were performed with BLAST program provided by NCBI. The phylogenetic tree was constructed from the evolutionary distance data calculated from Kimura's two-parameter model [32] using the neighbour-joining method [33].

### Expression and purification of the recombinant cellulase Cen219

The cellulase gene harbored in the clone cen219 was amplified by PCR with the pUC118-*cen219* as template with a *Bam*HI-linked sense primer P1 (5′-CGCGGATCCATGAACGCATTTCGTGGTGTGG-3′) and a *Hind*III-linked antisense primer P2 (5′-CCCAAGCTTGTGAGAACGTGCGCATGCG-3′). Amplified DNA was digested by *Bam*HI/*Hind*III, ligated into pET-30a which was linearized by *Bam*HI/*Hind*III, then transformed into *E. coli* BL21 cells. Transformed cells were then grown at 37°C in Luria-Bertani (LB) medium, supplemented with kanamycin (50 µg/ml) to a cell destiny of A_660_ = 0.4–0.6. Protein expression was induced by 1.0 mM IPTG (Sigma) and incubation was continued for 3 h at 37°C. The recombinant proteins were purified using purification protocol of 6× His-tagged proteins by Ni-NTA affinity chromatography according to pET System Manual. Cells were harvested by centrifugation at 1,066×g for 20 min and resuspended in lysis buffer (0.05 M NaH_2_PO_4_ [pH 8.0], 0.3 M NaCl, 0.02 M imidazole) and lysed by sonication. Cell debris were pelleted by centrifugation at 6,660×g for 30 min at 4°C and the supernatant was applied to a 2 ml column containing Ni-NTA resin. The concentration of the imidazole was increased to 0.04 M and the Ni-NTA resin was washed with 5 column volumes of buffer until the A_280_ was <0.01. Recombinant protein was eluted with the increasing imidazole to 0.25 M in the final wash. The final purified protein solution was desalted using a PD-10 ultrafiltration column (GE Healthcare, Mississauga, ON, Canada) by gravity flow according to the manufacturer's directions, eluted with 0.05 M citrate-phosphate buffer, pH 6.0 and analyzed by sodium dodecyl sulfate-polyacrylamide gel electrophoresis (SDS-PAGE). Protein concentration was determined by the method of Bradford [34] using bovin serum albumin (BSA) as a standard.

### Characterization analysis of Cen219

The cellulase activity assay was carried out using the method described by Miller et al. [35]. A 1.8 ml reaction mixture containing 0.5 µg of recombinant Cen219 with 1% CMC in 0.1 M pH 6.0 citrate phosphate buffer (appropriate diluted as needed) was incubated at 50°C for 20 min. The amount of reducing sugar released by hydrolysis was measured by 3, 5-dinitrosalicylate (DNS). One unit (U) of endoglucanase activity was defined as the amount of enzyme releasing 1 µmol of reducing sugar per min from the substrate. Cellulase activity was measured by the absorbance value of the mixture at 540 nm. The mixture without adding enzymes was as negative control.

Optimal temperature assay was determined by performing a standard activity assay in a temperature ranging from 30 to 70°C. To determine the enzyme stability at different temperatures, the residual activity of the Cen219 was assayed after incubation at different temperatures ranging from 30 to 70°C for 30 min.

To determine optimal pH, the enzyme activity assay was performed at different pH between 2 and 9. The pH stability was investigated by adding the purified Cen219 to buffers with different pH and incubating at 4°C for 24 h, followed by adjusting pH to 6.0 and then the residual activity was measured.

Substrate specificities of the enzyme were investigated using different kinds of substrates (oat gum, barley β-glucan, carboxymethyl cellulose, lichenan, oat spelt xylan, methyl cellulose, birchwood xylan, avicel and filter paper). The effect of several metal chloride salts (FeCl_3_, PhCl_2_, KCl, CaCl_2_, MgCl_2_, BaCl_2_, CuCl_2_, ZnCl_2_, CoCl_2_, HgCl_2_ and MnCl_2_) and chemicals (SDS, EDTA, 1, 10-phenanthroline hydrate, urea, Tween 80, Trixon X-100, *β*-Mercaptoethanol) was assayed by adding them into the reaction mixture and measuring the residual cellulase activity.

The kinetic constants, *K_m_* and *V_max_*, were calculated by directly fitting the data to the Michaelis–Menten equation by nonlinear regression. Reactions were carried out under optimal condition with CMC of different concentrations, ranging from 1 to 10 mg/ml.

All the activity assays above were performed in triplicate and the standard deviations were calculated.

## Results

### Construction and Screening of the Metagenomic Library

**Figure 1:**
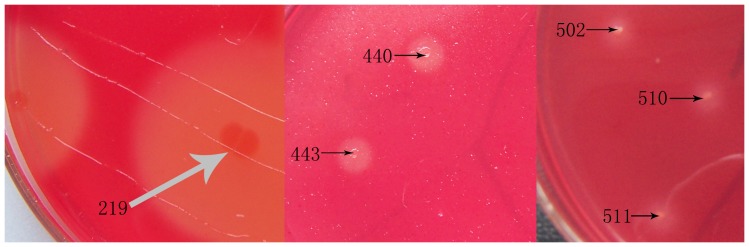
Screening result by congo red assay from the metagenomic library for CMCase activity, arrows indicated the clone names.

A metagenomic library containing about 5,000 clones was constructed from *Bx* and its associated microbe samples. To analyze the library quality, 20 clones were randomly chosen and the plasmids were digested by restriction enzymes. Restriction analysis showed that the inserted DNA fragments of these selected clones ranged from 1.5 to 5.5 kb with distinct restriction patterns, and the average insert size of these clones was estimated to be about 3.0 kb. Six positive clones (219, 440, 443, 502, 510, 511) expressing carboxymethyl cellulase (CMCase) activities were isolated and one clone named cen219 showed the maximum diameter of plate hydrolyzing ring (Fig. 1). Thus this clone with comparatively high cellulase production was selected for further study.

### Sequence Analysis of cellulase

**Figure 2:**
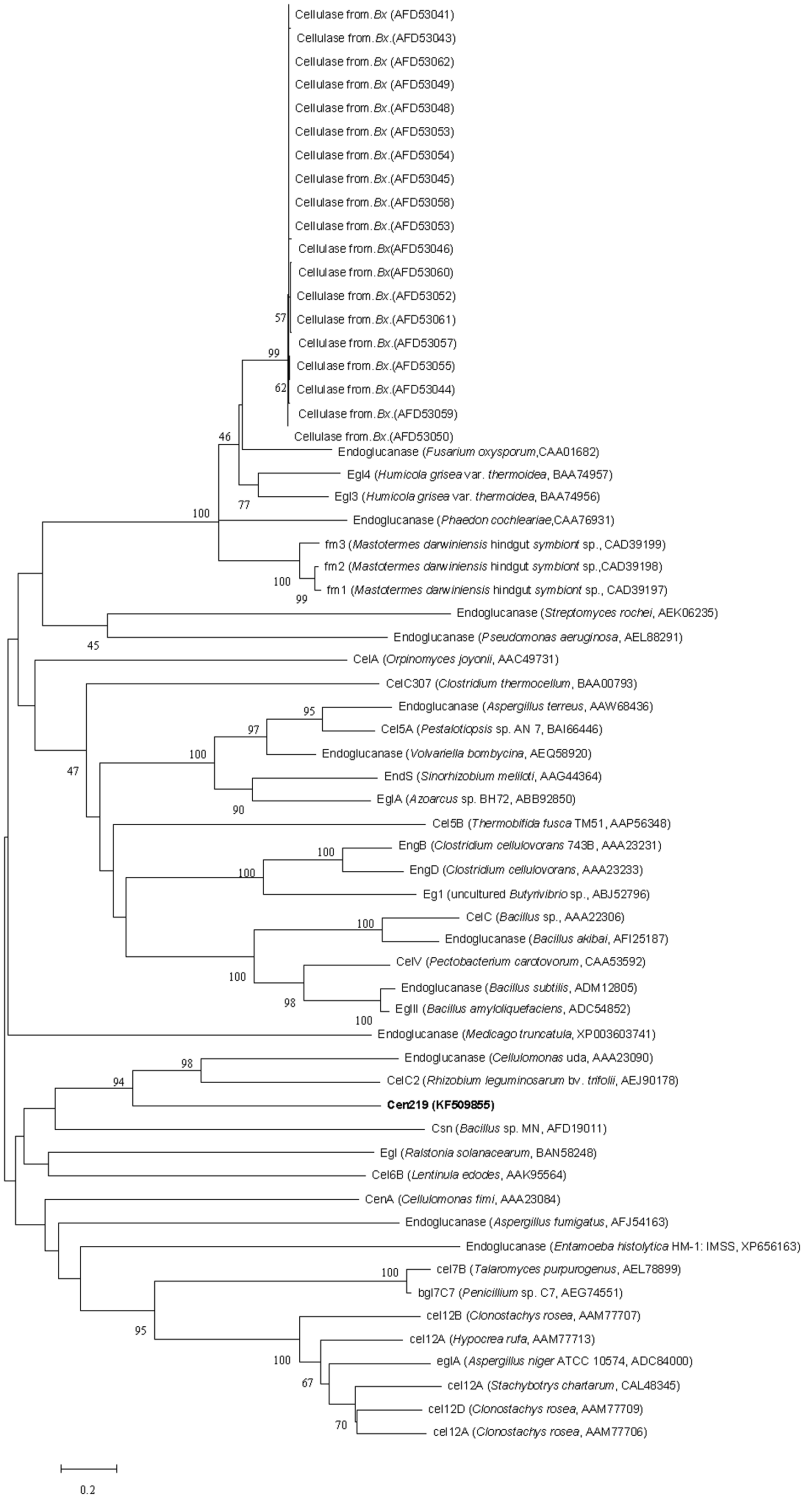
Phylogenetic tree analysis of endoglucanase superfamily homologous to Cen219 by neighbor-joining method.

The complete insert DNA sequence of cen219 was determined. The length of the insert DNA was 2,893 bp. BLAST analysis revealed the presence of an open reading frame consisting of 1,104 bp, encoding a fulllength endoglucanase gene (*cen219*). The gene *cen219* was submitted to GenBank (KF509855) and it encoded a protein of 367 amino acids with a predicted molecular mass of 40.66 kDa. The deduced amino acid sequence of Cen219 was used to perform a BLAST research of the NCBI and SwissProt databases. This search revealed that Cen219 belonged to the glycosyl hydrolase family 8 and the amino acid of the gene separately shared 90% with the endoglucanase III from *Klebsiella oxytoca*, and 87% with the endo-1, 4-D-glucanase from *Enterobacter aerogenes* KCTC 2190. The phylogenetic tree based on amino acid sequence was constructed to verify the evolutionary relationship of the Cen219 to other known endoglucanases, and 61 endoglucanase proteins including 19 from *Bx* were selected for the phylogenetic tree analysis. As shown in Figure 2, Cen219 is not closely related to other members of endoglucanase family, suggesting that Cen219 is a new member of endoglucanase.

### Expression and Purification of the Recombinant Cen219

**Figure 3:**
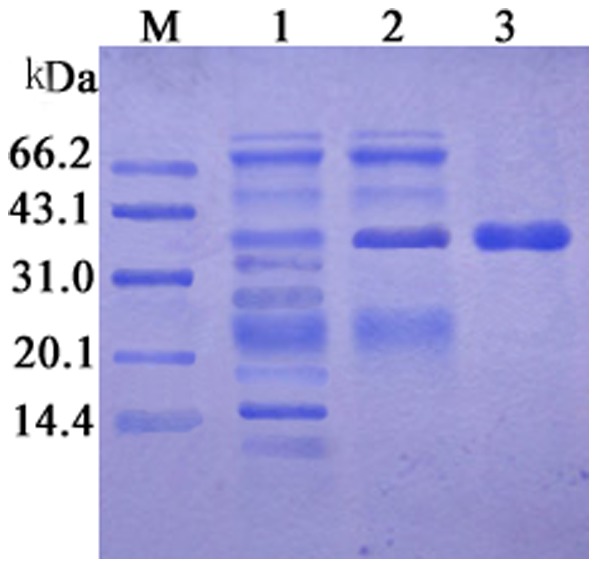
SDS-PAGE analysis of the purified recombinant Cen219. M, marker proteins; lane 1, extracts of IPTG-induced *E. coli* BL21 (pET-30a); lane 2, unpurified Cen219; lane 3, purified Cen219.

To characterize the biochemical properties of Cen219, *cen219* was expressed as an N-terminal His-tag fusion protein using pET-30a (t) expression system under the control of T7 lac promoter in *E. coli* BL21. No inclusion bodies were found in cell lysates after the cells were harvested and disrupted by sonication on ice, which suggested that the recombinant Cen219 was expressed in a soluble form. After purification with the Ni-NTA column, the molecular mass of the purified enzyme estimated by SDS-PAGE analysis was approximately 40 kDa (Fig. 3), which is consistent with our expectation. Therefore, the purified recombinant Cen219 was used to do the following analysis.

### Effect of pH and Temperature on Activity and Stability of Cen219

**Figure 4:**
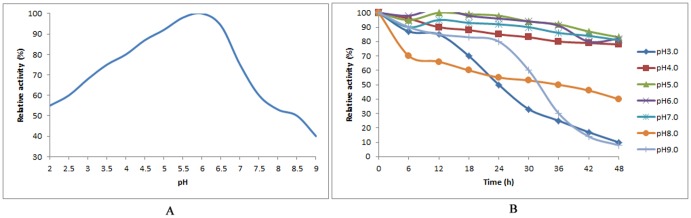
Effect of pH on activity and stability of Cen219. A. Effect of pH on activity of Cen219; B. Effect of pH on the stability of Cen219.

The results of pH studies indicated a broad pH activity range of 2.0–9.0 and optimum pH at 6.0 (Fig. 4). Less than 20% activity was retained at pH 10.0 after 20 min of incubation. The enzyme was stable at wide range of pH, still more than 80% activity was retained at pH 4–6 even after incubation for 24 h.

**Figure 5:**
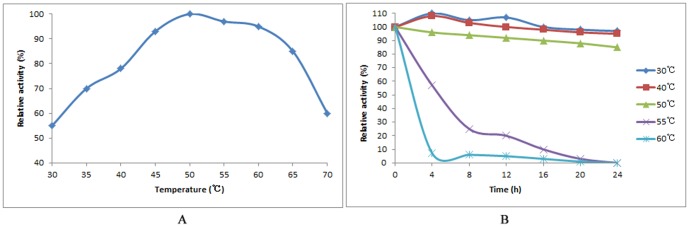
Effect of temperature on activity and stability of Cen219. A. Effect of temperature on activity of Cen219; B. Effect of temperature on the stability of Cen219.

Temperature optimization studies at pH 6.0 showed that at 50°C, the purified Cen219 had the maximum hydrolytic activity (Fig. 5). As for the stability of temperature, the purified enzyme still maintained about 90% activity below 50°C after 24 h of incubation, while when the temperature was above 55°C, the enzyme activity declined sharply. Almost no activity was detected at 60°C after 4 h of incubation.

### Effects of Different Chemicals on Cen219 Activity

The results of the effects of metal ions and chemicals were shown in table 1. Mn^2+^ enhanced the enzymatic activity to 148%, whereas Fe^3+^, Cu^2+^ and Hg^2+^ dramatically reduced enzyme activity to 19.7%, 22.9% and 36%, respectively. Co^2+^, Ca^2+^ and K^+^ slightly improved the activity. Ph^2+^, Mg^2+^, Zn^2+^ and Ba^2+^ had only slight inhibitory effects. The chelating agent EDTA and SDS had different inhibitory effects to the enzyme, and the enzyme activity was inhibited to 92.2% and 10.9% separately. While *β*-Mercaptoethanol did not alter activity and other chemicals all had slightly enhancing effects.

**Table 1:** Effects of metal ions, and chemicals on the enzyme activity of Cen219.

Metals	Concentrations	Rel act.(%) ± standard deviation
Fe^3+^	10 mM	19.7±2.2
Ph^2+^	10 mM	90.5±3.0
K^+^	10 mM	106.2±3.2
Ca^2+^	10 mM	102.1±3.0
Mg^2+^	10 mM	88.8±2.9
Ba^2+^	10 mM	74.1±2.0
Cu^2+^	10 mM	22.9±1.9
Zn^2+^	10 mM	83.6±3.7
Co^2+^	10 mM	105.5±3.8
Hg^2+^	10 mM	36±2.5
Mn^2+^	10 mM	148±3.8

Chemicals	Concentrations	Rel act.(%) ± standard deviation
SDS	1% w/v	10.9±1.8
EDTA	1 mM	92.2±2.9
1,10-phenanthroline hydrate	1 µM	108.3±3.4
urea	1 µM	109.6±3.5
Tween 80	1 µM	106.4±3.3
Trixon X-100	1 µM	104.4±2.7
*β*-Mercaptoethanol	1 µM	100.2±2.6

### Hydrolysis of Different Substrates

Substrate specificity of the recombinant Cen219 was determined under optimal conditions with 1% polysaccharides (Table 2). The enzyme showed the highest hydrolytic activity against barley oat gum. However, insoluble celluloses such as filter paper, avicel, birchwood xylan, methyl cellulose were hydrolyzed by the enzyme at considerably slower rate.

**Table 2:** Hydrolysis of various protein substrates by the purified Cen219.

Substrates	Specific activity^a^ (U/mg) ± SD	Rel act.(%)
Oat gum ((1–3,1–4)-β-D-glucan)	345.22±5.31	100
Barley β-glucan ((1–3,1–4)-β-D-glucan)	189.63±3.06	54.9
Carboxymethyl cellulose (1,4-β-D-glucan)	107.24±2.93	31.06
Lichenan ((1–3,1–4)-β-D-glucan)	82.44±2.07	23.9
Oat spelt xylan (1,4-β-D-xylan)	37.71±2.86	10.9
Methyl cellulose (1,4-β-D-glucan)	32.66±2.67	9.5
Birchwood xylan (1,4-β-D-xylan)	31.98±3.04	9.3
Avicel (1,4-β-D-glucan)	26.8±1.99	7.8
Filter paper	25.93±2.08	7.5

### Analysis of the kinetic constants of Cen219

**Figure 6:**
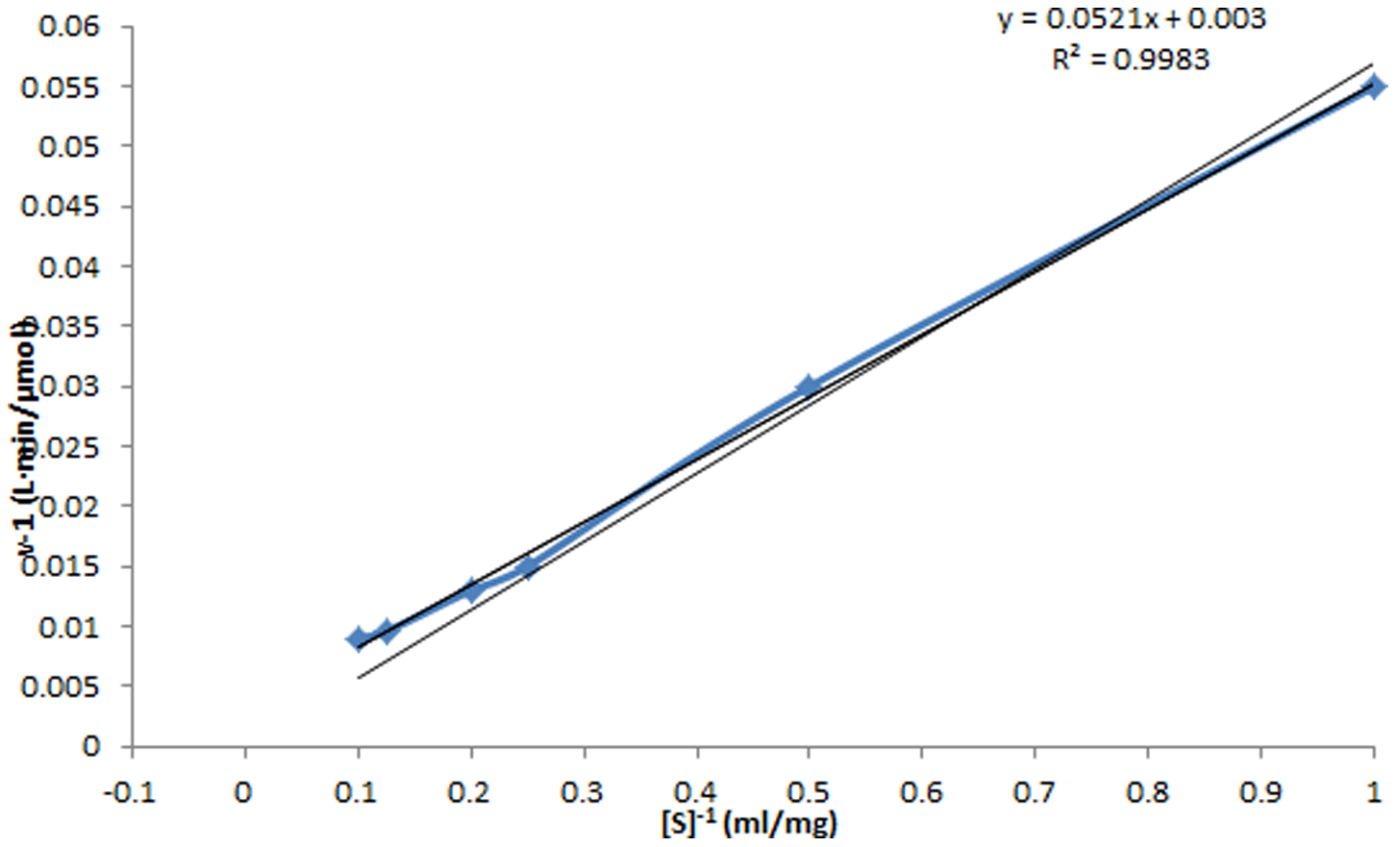
Kinetic constants determining for Cen219 towards CMC by Lineweaver-Burk assay.

The kinetic constants of Cen219 were measured by Lineweaver-Burk double reciprocal plot. According to the equation υ^−1^ = *K_m_*·*V_max_* ^−1^·[s]^−1^+*V_max_*, we can obtain a straight line via υ^−1^∼[s]^−1^ drawing. The intercept of x- coordinate and y-coordinate is respectively −*K_m_* ^−1^ and the *V_max_* ^−1^. As shown in Fig. 6, The *K_m_* and *V_max_* of the recombinant Cen219 towards CMC was 17.37 mg/ml and 333.33 U/mg, respectively.

## Discussion

Several cellulase genes (endo-β-1, 4-glucanase) have been identified from plant parasitic cyst and root-knot nematodes [36], [37]. These enzymes are produced within the oesophageal gland cells of the nematodes and are secreted through the nematode stylet into host tissues. Intriguingly, these genes appear to have been acquired by horizontal gene transfer from bacteria [18]. However, little is known about the abundant cellulase resources of industrial interest in *Bursaphelenchus* species. It is well-known that *Bx* is an important invasive plant parasitic nematode that has caused heavy mortality of trees in introduced regions including Japan, Korea, China, and Portugal [38], [39]. During the plant-feeding phase, the nematode *Bx* is usually characterized by rapid reproduction of the nematode and tree death. Therefore *Bx* has a close association with plant. Since cellulose is the main structural component of plant cell walls, it seems that cellulases play an important role in the life cycle of these nematodes. Since majority of the microorganisms in nature cannot be cultured, it limits the discovery of novel biocatalysts. To overcome this limitation, metagenomics can be an alternative to access to the unculturable microorganisms. In this study, we constructed a metagenomic library with the sample from China to explore novel cellulases. Out of approximately 5,000 clones, six CMCase positive clones were obtained. Among which, the clone cen219 with the maximum cellulase activity was further identified as a novel cellulase gene via analysis of the nucleotide and amino acid sequence. To our knowledge, this is the first report about cellulases from *Bx* through metagenomic library by functional screening.

The gene *cen219* from metagenomic library was cloned and expressed in *E. coli*, and its product was purified to be characterized. The molecular masse of cellulases varies a wide range of sizes. The minimum of the cellulase is 6.3 kDa from *Cytophaga* [40], while the cellulase from *Fusarium solani* is up to 400 kDa, which is the maximum size [41]. The purified Cen219 is 40 kDa, which was different from some known endoglucanases with molecular masses ranging from 35.9 to 659 kDa [42], [43]. The optimum temperature of 50°C was comparable to that recorded from dairy cow rumen [12], insect gut bacterium [44] and a *Bacillus* strain isolated from cow dung [45]. Cen219 is active over a wide range of pH, retaining 40% of its activity even at pH 9.0, and it is stable even at pH 4.0–6.0. The endoglucanase Cen219 was only partially inhibited when incubated in 1 mM EDTA, suggesting that Cen219 does not absolutely require divalent cations to degrade CMC. Furthermore, addition of 1.0 mM Ca^2+^ or Co^2+^ to the incubation buffer slightly stimulated activity, which is consistent with several family 9 glycoside hydrolases [46], [47]. As with the most cellulases, the metal ion Mn^2+^ greatly enhanced the activity of Cen219 [48]–[50]. It has been reported that metallic ions, such as Fe^3+^, Cu^2+^, Zn^2+^, exert an inhibitory effect on family 5 glycoside hydrolases [47], [51]. As for the enzyme Cen219, Fe^3+^ and Cu^2+^ showed strong inhibitory, while Zn^2+^ had slightly inhibitory effect. Analysis of the utilization of substrates indicated that the Cen219 hydrolyze easier β-(1, 4)-linkage polysaccharides than β-(1, 3)-linkage ones. Cen219 showed the highest activity against mixed-linkage β-glucans from barley and oats but lower against the mixed-linkage β-glucan from lichenan (23.9%). The reason for that is probably the higher proportion of β-(1, 3)-linkages in lichenan. Microcrystalline cellulose avicel and filter paper was hydrolyzed by Cen219 at considerably the slowest rate, which is different with the reported other cellulases that cannot hydrolyze those substrates [47], [52]. The broad substrate specificity of Cen219 is consistent with previous reports on cellulases from other glucanase families [53]–[58].

In conclusion, a novel endoglucanase family 8 glycoside hydrolase was successfully isolated from a metagenomic library with the sample from PWN *Bx* by functional screening and expressed in *E. coli*. The recombinant Cen219 was purified and characterized. The enzyme exhibited moderate thermostability and wide-range pH stability. It is with significant activity towards a variety of β-1, 4- and β-(1, 3-1, 4) glucans, including microcrystalline and filter paper celluloses. The properties made Cen219 a potential candidate for biological applications. Further studies of directed evolution and three-dimensional structure are expected to explain the uniqueness of Cen219, and the comparison of Cen219 with commercial fungal cellulases (e.g., from *Trichoderma reesei*) is necessary to evaluate the cellulase's industrial application. The meaning of the findings is to lay a theoretical foundation for constructing high-level expression strains. Moreover, our results in this work would help understanding the nature of the molecules produced by nematode *Bx* that allow them to parasitize plants.
